# Are Interferon-Free Direct-Acting Antivirals for the Treatment of HCV Enough to Control the Epidemic among People Who Inject Drugs?

**DOI:** 10.1371/journal.pone.0143836

**Published:** 2015-12-03

**Authors:** Viviane D. Lima, Ignacio Rozada, Jason Grebely, Mark Hull, Lillian Lourenco, Bohdan Nosyk, Mel Krajden, Eric Yoshida, Evan Wood, Julio S. G. Montaner

**Affiliations:** 1 British Columbia Centre for Excellence in HIV/AIDS, Vancouver, British Columbia, Canada; 2 The Kirby Institute, University of New South Wales Australia, Sydney, NSW, Australia; 3 Public Health Microbiology and Reference Laboratory, British Columbia Centre for Disease Control, Vancouver, British Columbia, Canada; 4 Division of Gastroenterology, Department of Medicine, Faculty of Medicine, University of British Columbia, Vancouver, British Columbia, Canada; University of Alberta, CANADA

## Abstract

**Background:**

Widely access to interferon-free direct-acting antiviral regimens (IFN-free DAA) is poised to dramatically change the impact of the HCV epidemic among people who inject drugs (PWID). We evaluated the long-term effect of increasing HCV testing, treatment and engagement into harm-reduction activities, focused on active PWID, on the HCV epidemic in British Columbia (BC), Canada.

**Methods:**

We built a compartmental model of HCV disease transmission stratified by disease progression, transmission risk, and fibrosis level. We explored the effect of: (1) Increasing treatment rates from 8 to 20, 40 and 80 per 1000 infected PWID/year; (2) Increasing treatment eligibility based on fibrosis level; (3) Maximizing the effect of testing by performing it immediately upon ending the acute phase; (4) Increasing access to harm-reduction activities to reduce the risk of re-infection; (5) Different HCV antiviral regimens on the Control Reproduction Number *R*_*c*_. We assessed the impact of these interventions on incidence, prevalence and mortality from 2016 to 2030.

**Results:**

Of all HCV antiviral regimens, only IFN-free DAAs offered a high chance of disease elimination (i.e. *R*_*c*_ < 1), but it would be necessary to substantially increase the current low testing and treatment rates. Assuming a treatment rate of 80 per 1000 infected PWID per year, coupled with a high testing rate, the incidence rate, at the end of 2030, could decrease from 92.9 per 1000 susceptible PWID per year (Status Quo) to 82.8 (by treating only PWID with fibrosis level *F*_2_ and higher) or to 65.5 (by treating PWID regardless of fibrosis level). If PWID also had access to increased harm-reduction activities, the incidence rate further decreased to 53.1 per 1000 susceptible PWID per year. We also obtained significant decreases in prevalence and mortality at the end of 2030.

**Conclusions:**

The combination of increased access to HCV testing, highly efficacious antiviral treatment and harm-reduction programs can substantially decrease the burden of the HCV epidemic among PWID. However, unless we increase the current levels of treatment and testing, the HCV epidemic among PWID in BC, and in other parts of the world with similar epidemiological background, will remain a substantial public health concern for many years.

## Introduction

The burden of hepatitis C virus (HCV) infection remains a major public health challenge, particularly among people who inject drugs (PWID), who have an estimated global HCV prevalence of 67% [1]. Studies have shown that PWID acquire HCV relatively fast following initiation of injecting illicit drugs (2). Given that there is no efficacious preventive HCV vaccine on the horizon, engagement in harm-reduction programs (specifically the use of needle exchange, drug treatment programs (pharmacological or not), and supervised injection sites), timely HCV diagnosis, assessment and treatment for HCV infection are key components for decreasing HCV-related disease burden [2–4]. Without treatment, 15%-25% of chronically infected individuals will develop cirrhosis, with concomitant increased risk for end-stage liver disease and hepatocellular carcinoma [5]. To date, there is still a large number of individuals unaware that they are infected with HCV (50% in the United States, >60% in Europe and 26% in cohort studies in Vancouver Canada), and similarly, a large number of individuals with no or limited access to treatment (<10% in the United States and Canada and <16% in Europe); thus, ongoing HCV transmission remains a concern [6–12].

Until recently, those infected with HCV were treated with pegylated interferon and ribavirin (pegIFN–RBV) for 24 to 48 weeks depending on the HCV genotype [5]. The primary goal of treatment is to achieve sustained virologic response (SVR); i.e. to achieve a virological cure defined as no detectable HCV in the bloodstream 24 weeks after treatment [5]. Since the pegIFN–RBV treatment had poor efficacy and associated safety and tolerability issues, its uptake was extremely low. Since 2011, the HCV treatment field has been revolutionized with the approval of higher-efficacy (although poorly tolerated[13]) drugs such as boceprevir (BOC) and telaprevir (TPV), and more recently sofosbuvir, which is more tolerable and with treatment efficacies as high as 90% for most common HCV genotypes [14–17].

The advent of IFN-free regimens of direct-acting agents (DAAs) is poised to dramatically change the HCV treatment landscape. In recent randomized clinical trials, these agents have markedly increased therapeutic efficacy due to their higher potency, improved safety profile and ease of administration [18]. In 2015, a highly tolerable and efficacious DAA combination treatment of ledipasvir and sofosbuvir was approved by Health Canada for people with genotype 1 hepatitis C virus [19]. It is anticipated that fully oral, once daily regimens with pan-genotypic activity will be able to eradicate HCV infection within 12 weeks in more than 95% of patients [20].

In view of these therapeutic advances, recent mathematical models have demonstrated the dramatic effect that increasing HCV treatment coverage has on decreasing both HCV incidence and prevalence, particularly when using IFN-free DAA regimens [13, 21–25]. Based on these advances in HCV treatment, we propose to evaluate the potential impact of increasing HCV testing, treatment and engagement into harm-reduction activities, focused on active PWID, on the HCV epidemic in British Columbia (BC), Canada. The ultimate goal of this model is to provide theoretical support for the implementation of a combination of these previous strategies aimed at reducing the individual and public health burdens of the HCV epidemic amongst active PWID not only in BC, but also around the world.

## Materials and Methods

The use of mathematical modeling in this study provides a means to combine the complex individual-based knowledge of clinical, behavioural and epidemiological aspects of the HCV epidemic. Based on the premise of this type of model, we are able to forecast epidemic trends, determine key factors that influence the course of infection within and between individuals, and predict the impact of different interventions such as the ones investigated in this study.

### Deterministic Compartmental Transmission Model

**Fig 1:**
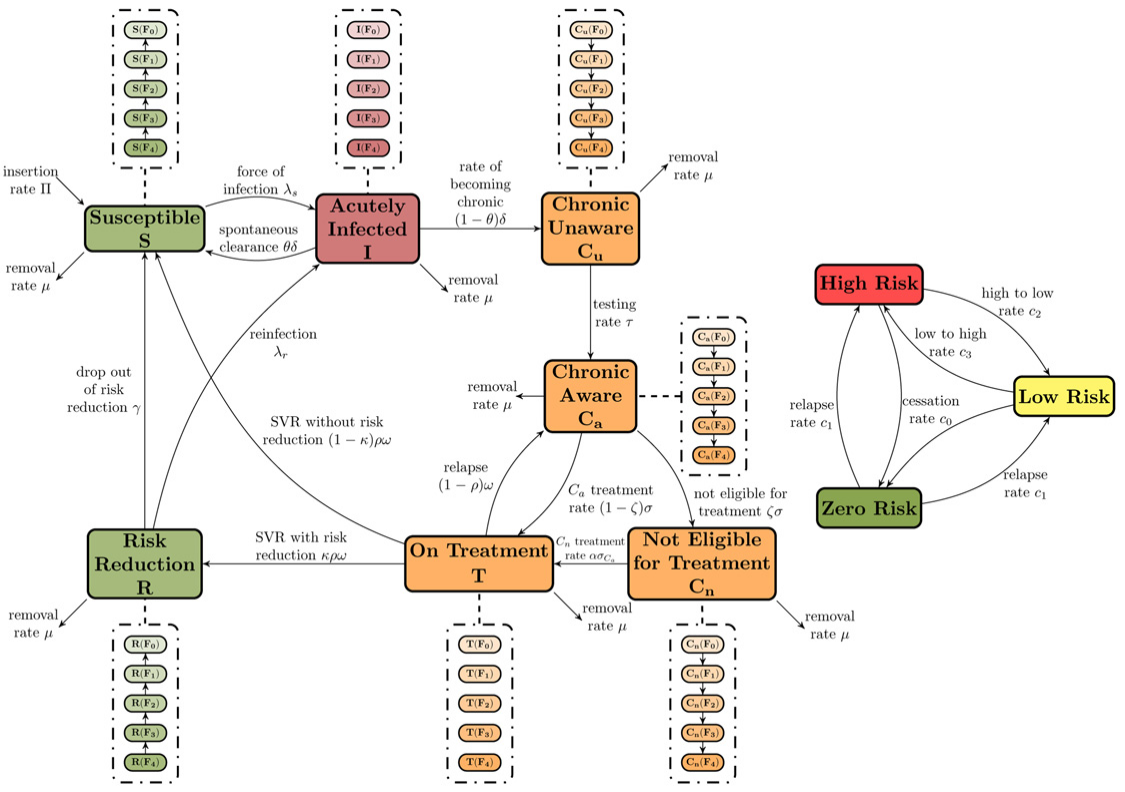
HCV Transmission and Disease Progression Model Schematic.

The model has seven mutually exclusive compartments (Fig 1): (1) Susceptible (*S*)—individuals at-risk of HCV infection among those never exposed to the HCV virus or at risk of being re-infected among those virus-experienced who achieved SVR; (2) Risk reduction (*R*)—individuals at-risk of re-acquiring HCV among virus-experienced individuals. These individuals have achieved SVR, and instead of going back to the “pool” of susceptibles, they receive access to increased harm-reduction activities to reduce their risk of re-infection; (3) Acutely Infected (*I*)—individuals who have recently become newly infected or re-infected; (4) Chronic Unaware (*C*_*u*_)—after the acute phase, individuals enter the chronic stage and stay in this compartment until they get tested for HCV; (5) Chronic Aware (*C*_*a*_)—there are two sets of individuals in this compartment: those who had been tested (confirmed to be HCV RNA positive) during the chronic stage and were waiting for treatment, and individuals who previously failed treatment and were still eligible to receive treatment; (6) On Treatment (*T*); and (7) Chronic aware and not eligible for treatment (*C*_*n*_)—individuals who have been deemed ineligible for treatment due to contraindication and environmental reasons (e.g., unstable housing, ongoing drug use).

Each of these compartments has sub-compartments corresponding to the five liver fibrosis stages in the METAVIR scoring system [26]—*F*_0_ (i.e., no fibrosis) to *F*_4_ (i.e., cirrhosis) (Fig 1). We assumed that individuals while in the *I*,*C*_*u*_,*C*_*a*_ and *C*_*n*_ compartments can sustain increasing liver damage and move from fibrosis stage *F*_*j*_ to *F*_*j*+1_, for *j* = 0,1,2,3 depending on the time they spend in these stages; and individuals in the *S* and *R* compartments can reduce their liver damage (due to treatment) and move from fibrosis stage *F*_*j*_ to *F*_*j*−1_ for *j* = 0,1,2,3 also based on the time these individuals spend on these compartments. Since there is no population-level data regarding the fibrosis distribution amongst PWID in BC, the fibrosis distribution for this model was numerically derived to fit the BC prevalence, which is estimated to be 65% of the PWID population. In other words, with all model parameters fixed, after a long-time simulation, this model arrived at the endemic equilibrium, which gave us the baseline distribution of fibrosis amongst the PWID population in BC.

In this model, at each interaction, PWID were classified based on three risk levels: two injection risk scenarios (high and low risk) based on whether PWID access harm-reduction services, and an additional zero-risk scenario where PWID can temporarily cease injection drug use (approximately after 5.7 years) [27]. Additionally, PWID can exit the model via mortality; the mortality parameter for compartments involving HCV-infected PWID was specific to their fibrosis level. Throughout the simulation, some HCV-infected PWID become unable to transmit HCV by temporary cessation of injection drug use, and they return to be able to transmit HCV by relapse into injection drug use (approximately after 1.3 years)[27]. Additionally, the number of PWID tested and treated throughout the simulation was obtained by multiplying the testing (*τ*) or treatment (*σ*) rates by the number of chronically unaware (i.e. *C*_*u*_ compartment) or aware (i.e. *C*_*a*_ compartment) PWID, respectively. Hence, this formulation guarantees that we never tested or treated more individuals than those available in those compartments.

This model was calibrated based on available data to match the 2012 HCV prevalence in BC (i.e. 18,068 PWID in BC, assuming that 65% are HCV RNA positive [22]). This calibration was based on the parameter *β* (i.e. the average contact rate between susceptible and infected PWID), which was numerically derived to fit the 65% HCV prevalence in BC in the endemic scenario. As such, with all other parameters fixed, we ran a long-time simulation inside a Nelder-Mead simplex algorithm that determined the optimal value of *β* assuming a tolerance of 10^−6^ [28]. These analyses were implemented using the optimization package in the SCIPY library in Python^TM^ version 2.7.6.

The Status Quo scenario was for the stable endemic equilibrium, which was reached under the constraints of keeping a constant total PWID population (N = 18,068) and HCV prevalence (65%) throughout the simulation, using Peg-IFN+RBV treatment as the standard of care up to 2012 in BC. We decided to use data from 2012 to start our simulation, since we do not have more current information on testing and treatment uptake in BC. We then let the model run from 2013 to 2030 adjusting for changes in treatment guidelines in which Peg-IFN+RBV plus (BOC or TPV) is the current standard of care in BC since 2013. It is important to mention that throughout all simulated scenarios, we kept the total PWID population (N = 18,068) constant.

In addition, based on available testing and clinical data in BC from 2003 to 2009, we assumed that the HCV genotype distribution in our population was 65% for genotype 1, 30% for genotype 2 and 3 and 5% for other genotypes, and that, yearly, approximately 140 per 1000 infected PWIDs are tested and that 8 per 1000 infected PWID are treated for HCV [8]. Note that based on the current standard of care in BC, eligible individuals need to have fibrosis level *F*_2_ and higher to be eligible for treatment.

All model parameters and their explanation along with the set of differential equations can be found in the S1 File. The differential equation model was implemented using the scientific computing libraries in Python^TM^ version 2.7.6.

### Main Outcomes

We projected the course of the HCV epidemic in BC by comparing the Status Quo scenario to different HCV-TTHR scenarios. The measures of intervention impact at the end of 15 years (from 2016 until 2030) included: (1) HCV incidence rates—obtained by dividing the estimated number of new HCV cases by the estimated size of the susceptible PWID population in a given calendar year; (2) all-cause mortality rates—obtained by dividing the estimated number of deaths by estimates of the size of the infected PWID population in a given calendar year; and (3) HCV prevalence—obtained by dividing the estimated number of individuals living with HCV by the estimated size of the PWID population in a given calendar year. We ultimately presented these outcomes in terms of percent change in incidence, prevalence and mortality at the end of 2030. We calculated the percentage change in each of these outcomes when comparing the different intervention scenarios to the Status Quo scenario.

We also calculated the effect of the four HCV antiviral regimen scenarios on the Control Reproduction Number (*R*_*c*_) for a wide range of treatment and testing rates. The definition of stable endemic equilibrium can be in terms of *R*_*c*_ [29], which assumes that the entire population is susceptible and biomedical and/or behavioral interventions are in place [30]. A *R*_*c*_ < 1 means that, over time, the model will reach the disease-free equilibrium—meaning that on average an infected individual produces less than one new infection over the course of his/her infectious period, and thus, the epidemic cannot grow. A *R*_*c*_ > 1 means that, over time, the model will reach a stable endemic equilibrium—meaning that each infected individual produces, on average, more than one new infection, and the disease continues to spread in the population.

### Modeling Scenarios

In this model, from 2016 until 2030, we assessed the effect of the following interventions:

#### Treatment and testing

We increased treatment coverage rates from 8 to 20, 40 and 80 per 1000 PWID per year, assuming that IFN-free DAA becomes the standard of care from 2016 to 2030 (Intervention Scenario 1). Based on the conditions in the Intervention Scenario 1, we additionally assessed the effect of increasing treatment coverage and eligibility to all individuals regardless of fibrosis level (Intervention Scenario 2). At last, based on the conditions in the Intervention Scenarios 1 and 2, we explored a best case scenario in which testing is done immediately upon ending the acute phase to assess the maximal effect that increased testing can have on controlling this epidemic (Intervention Scenario 3).

#### Treatment and testing conditions for the elimination of the HCV epidemic

We assessed the effect of different HCV antiviral regimens on *R*_*c*_ if the infected PWID in the population were receiving: (i) Peg-IFN+RBV which was the standard of care in BC up to 2012—we assumed a 55% SVR and a duration of treatment between 24–48 weeks depending on the genotype; (ii) Peg-IFN+RBV plus (BOC or TPV) which is the current standard of care in BC—we assumed a 70% SVR and a duration of treatment between 24–48 weeks depending on the genotype; (iii) IFN-free DAA which is expected to be the standard of care in one to two years—we assumed a 90% SVR and a duration of treatment of 12 weeks (conservative assumption) regardless of genotype; and (iv) the ‘Best Case’ scenario in which we assumed a 100% SVR and a duration of treatment of 1 week regardless of genotype.

#### Engagement into harm-reduction activities

This last set of interventions relates to the engagement of individuals into harm-reduction activities to reduce their risk of being re-infected after achieving SVR. Note that in the model, the parameter *β* (i.e. the average contact rate between susceptible and infected individuals) includes the background rate of individuals in the population currently accessing harm-reduction programs (53 per 100 PWID per year) [31], and this parameter was fit to match the BC prevalence. This intervention was modeled by including a compartment (*R*) in which some individuals, upon achieving SVR, receive access to increased harm-reduction activities instead of returning to the pool of susceptibles. The rate in which these individuals move into the *R* compartment is given by the parameter *κ*. In the baseline scenario, we assumed that 0 per 1000 newly-cured PWID go into this compartment since it is one of our proposed interventions and it is not currently available in BC; in the intervention scenarios we assumed that 100, 200, 400, 500, 600 and 800 per 1000 newly-cured PWID per year go into this compartment after achieving SVR. Note that the removal rate (*γ*) from this compartment and into the Susceptibles compartment was assumed to be constant and set at 60 per 1000 PWID per year [32].

### Sensitivity analysis

We first estimated the univariate sensitivity coefficients for the incidence and prevalence changes under the three treatment uptake scenarios (i.e. increase treatment rate from 8 to 20, 40 and 80 per 1000 PWID per year) for the top ten parameters at the end of 2030. These sensitivity coefficients measure the relative change in the target variable with respect to the relative change in a model parameter [33]. Positive coefficients occur when changes (i.e. an increase or a decrease) in a parameter are positively associated with changes in the target variable (i.e. HCV incidence rate or prevalence). Negative coefficients occur when changes in a parameter are negatively associated with changes in the target variable. The magnitude of the sensitivity coefficient reflects how sensitive the target variable is to changes in each parameter. The sensitivity analyses were implemented using the scientific computing libraries in Python^TM^ version 2.7.6.

Secondly, we estimated the percent change in HCV incidence rate and prevalence at the end of 2030, with respect to our model predictions based on the Status Quo scenario, with the exception that we increased treatment coverage to 80 per 1000 PWID treated per year and DAA was modeled as the standard of care from 2016 to 2030. We considered lower and higher values for the parameters with most uncertainty based on the available literature: fibrosis regression time (4 versus 13.3 (as in [34]) years), fibrosis progression time (9.6 (as in [35]) versus 21.2 (as in [36]) years), average treatment length (8 versus 16 weeks), SVR rate (80% versus 100%), high versus low infectivity (2.0/0.4 (as in [22]) versus 1/1), time to relapse into injection drug use (5 versus 1 year), injection drug use duration (10 versus 4 years), relative infectivity while chronically infected with respect to the acutely infected stage (20% (as in [22]) versus 7%), and the assumed HCV prevalence in BC at the Status Quo scenario (50% versus 75%).

### Ethics approval

This study received approval from the University of British Columbia ethics review committee at the St Paul’s Hospital, Providence Health Care site (H13-00469). Note that all data collected for the model is publicly available online through different published manuscripts and reports. Thus, we did not utilize any patient-level information to conduct this study and there was no need to obtain informed consent.

## Results

### Status Quo

The model predicted that between 2016 and 2030, there would be an estimated 8,832 cumulative new HCV infections and 5,169 cumulative number of mortality cases. Additionally, the model estimated that the HCV incidence rate in 2030 would be 92.9 per 1000 susceptible PWID per year and the mortality rate would be 29.4 per 1000 infected PWID per year. Note that in the Status Quo scenario, the HCV prevalence was kept constant at 65% of the PWID population.

### Effect of testing and treatment

**Fig 2:**
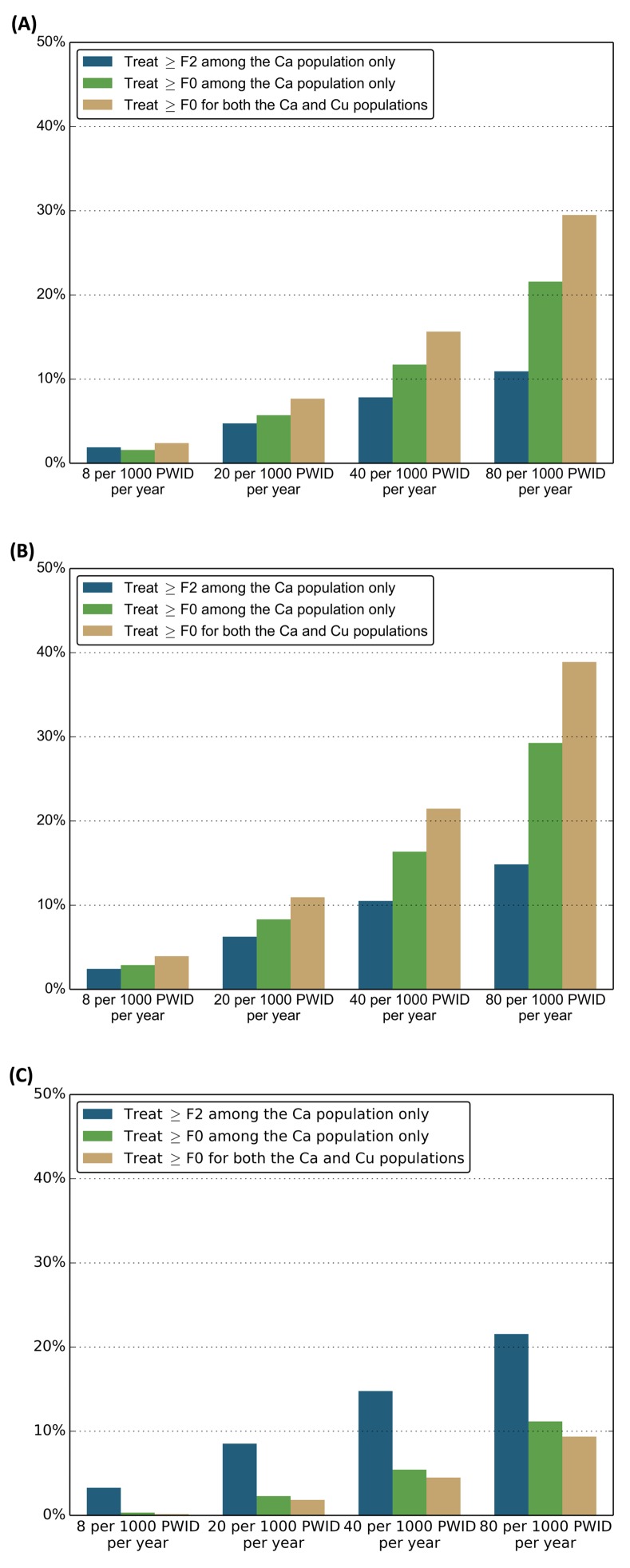
Percent change in (A) HCV incidence rate, (B) HCV prevalence and (C) mortality, at the end of 2030, for different testing and treatment scale-up scenarios. Blue bars: assume that 140 per 1000 PWIDs are tested and that 8, 20, 40 and 80 per 1000 PWID are treated for HCV per year; treatment eligibility applies only to the chronic aware population (*C*_*a*_) with fibrosis level *F*_2_ and higher. Green bars: assume that 140 per 1000 PWIDs are tested and that 8, 20, 40 and 80 per 1000 PWID are treated for HCV per year; treatment eligibility applies only to the chronic aware population (*C*_*a*_) with fibrosis level *F*_0_ and higher. Gold bars: assume that PWIDs are tested immediately upon ending the acute phase and that 8, 20, 40 and 80 per 1000 PWID are treated for HCV per year; treatment eligibility applies both to the chronic unaware (*C*_*u*_) and aware (*C*_*a*_) population with fibrosis level *F*_0_ and higher. Note that in this case PWID do not spend time in the *C*_*u*_ compartment and go straight to the *C*_*a*_ compartment due to the testing intervention.

Fig 2 presents the results for the percent change in the estimated HCV incidence rate, prevalence and mortality rate, in comparison to the Status Quo scenario at the end of 2030.

#### Intervention Scenario 1

When we changed the type of antiviral treatment from Peg-IFN+RBV plus (BOC or TPV) to INF-free DAA, the estimated HCV incidence rate (per 1000 susceptible PWID per year) decreased to 91.1 (1.9% decrease), 88.5 (4.7% decrease), 85.6 (7.8% decrease) and 82.8 (10.9% decrease) for the treatment scenarios 8, 20, 40 and 80 PWID treated per year, respectively (Fig 2(A) blue bar). The estimated HCV prevalence decreased to 63% (2.4% decrease), 61% (6.2% decrease), 58% (10.5% decrease) and 55% (14.8% decrease) for the treatment scenarios 8, 20, 40 and 80 PWID treated per year, respectively (Fig 2(B) blue bar). The estimated HCV mortality rate (per 1000 infected PWID per year) decreased to 28.4 (3.3% decrease), 26.9 (8.5% decrease), 25.0 (14.8% decrease) and 23.0 (21.5% decrease) for the treatment scenarios 8, 20, 40 and 80 PWID treated per year, respectively (Fig 2(C) blue bar).

#### Intervention Scenario 2

In addition to the conditions in the Intervention Scenario 1, here we changed treatment eligibility (i.e. fibrosis *F*_0_ and higher). The estimated HCV incidence rate decreased to 91.4 (1.6% decrease), 87.6 (5.7% decrease), 82.0 (11.7% decrease) and 72.8 (21.6% decrease) for the treatment scenarios 8, 20, 40 and 80 PWID treated per year, respectively (Fig 2(A) green bar). The estimated HCV prevalence decreased to 63% (2.9% decrease), 59% (8.3% decrease), 54% (16.3% decrease) and 46% (29.3% decrease) for the treatment scenarios 8, 20, 40 and 80 PWID treated per year, respectively (Fig 2(B) green bar). The estimated HCV mortality rate (per 1000 infected PWID per year) decreased to 29.3 (0.3% decrease), 28.7 (2.3% decrease), 27.8 (5.4% decrease) and 26.1 (11.2% decrease) for the treatment scenarios 8, 20, 40 and 80 PWID treated per year, respectively (Fig 2(C) green bar).

#### Intervention Scenario 3

Based on the conditions in the Intervention Scenarios 1 and 2, if we also increased testing immediately upon ending the acute phase, the estimated HCV incidence rate decreased to 90.7 (2.4% decrease), 85.8 (7.7% decrease), 78.4 (15.6% decrease) and 65.5 (29.5% decrease) for the treatment scenarios 8, 20, 40 and 80 PWID treated per year, respectively (Fig 2(A) gold bar). The estimated HCV prevalence decreased to 62% (3.9% decrease), 58% (10.9% decrease), 51% (21.5% decrease) and 40% (38.9% decrease) for the treatment scenarios 8, 20, 40 and 80 PWID treated per year, respectively (Fig 2(B) gold bar). The estimated HCV mortality rate (per 1000 infected PWID per year) decreased to 29.3 (0.2% decrease), 28.8 (1.8% decrease), 28.0 (4.5% decrease) and 26.6 (9.4% decrease) for the treatment scenarios 8, 20, 40 and 80 PWID treated per year, respectively (Fig 2(C) gold bar).

#### Effect of interventions on the distribution of fibrosis level

**Fig 3:**
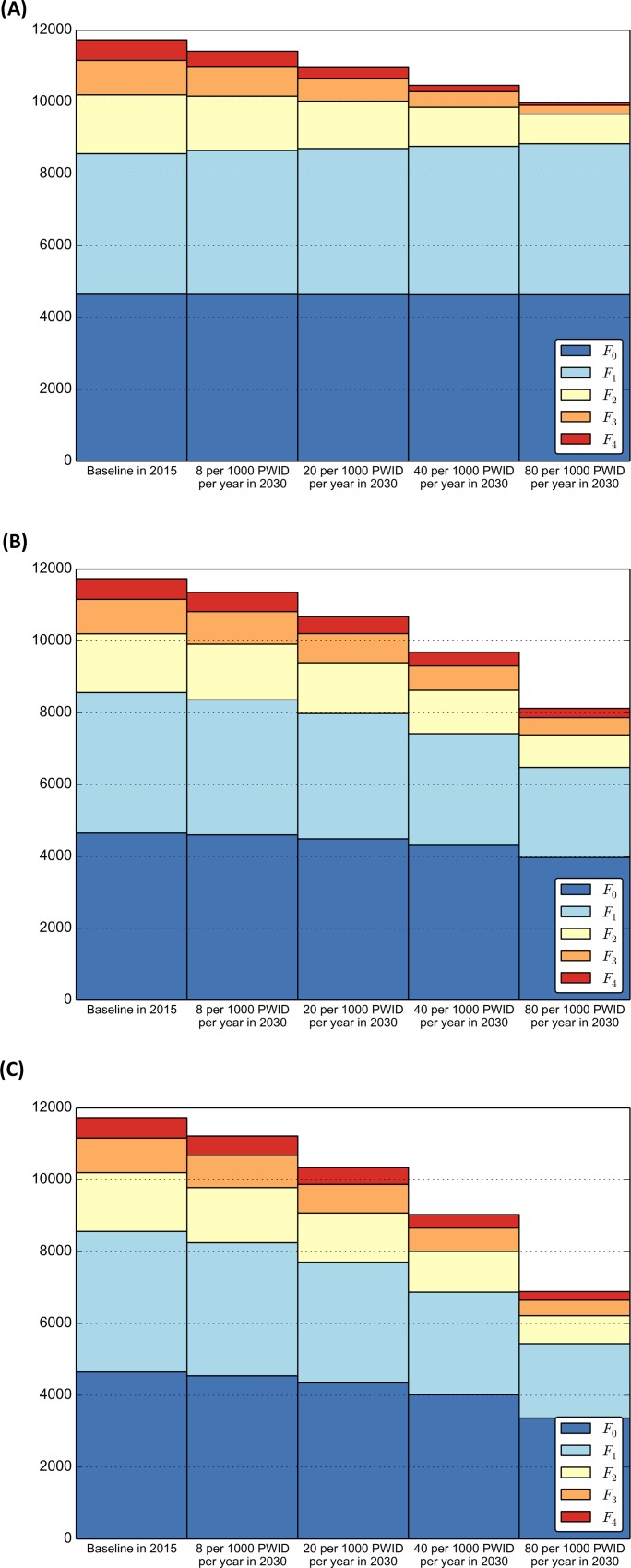
Model results for the estimated number of HCV prevalent cases (y-axis) at the end of 2030 stratified by fibrosis level (*F*_0_,…,*F*_4_) and treatment scale-up scenarios; (A) Based on the current standard of care in British Columbia for HCV treatment eligibility (i.e. fibrosis level *F*_2_ and higher) (Intervention Scenario 1); (B) Based on a broad treatment eligibility in which PWID are treated regardless of their fibrosis level (i.e. fibrosis level *F*_0_ and higher). In all scenarios individuals received IFN-free DAAs (Intervention Scenario 2); (C) Based on a broad treatment eligibility in which PWID are treated regardless of their fibrosis level (i.e. fibrosis level *F*_0_ and higher) and higher testing rates (i.e. immediately upon ending the acute phase) (Intervention Scenario 3). In all scenarios individuals received IFN-free DAAs.

Fig 3 presents the results for the estimated HCV prevalence for Intervention Scenarios 1 to 3 in comparison to the Status Quo scenario at the end of 2030.

Based on Fig 3(A), the distribution of the two lowest fibrosis levels (*F*_0_ and *F*_1_) remains relatively unchanged since these individuals need to wait to become eligible to receive treatment. Only when we treated individuals regardless of their fibrosis level (Fig 3(B)), we observed a stronger shift in the fibrosis distribution in the higher treatment scenario in comparison to the baseline scenario. This effect was even more noticeable when we also increased the testing rate (Fig 3(C)), in which testing was done immediately upon ending the acute phase to assess the maximal effect that increased testing can have on controlling this epidemic. Please see S1 File for a detailed analysis of the results presented in Fig 3.

### Treatment and Testing conditions for the Elimination of the HCV Epidemic

**Fig 4:**
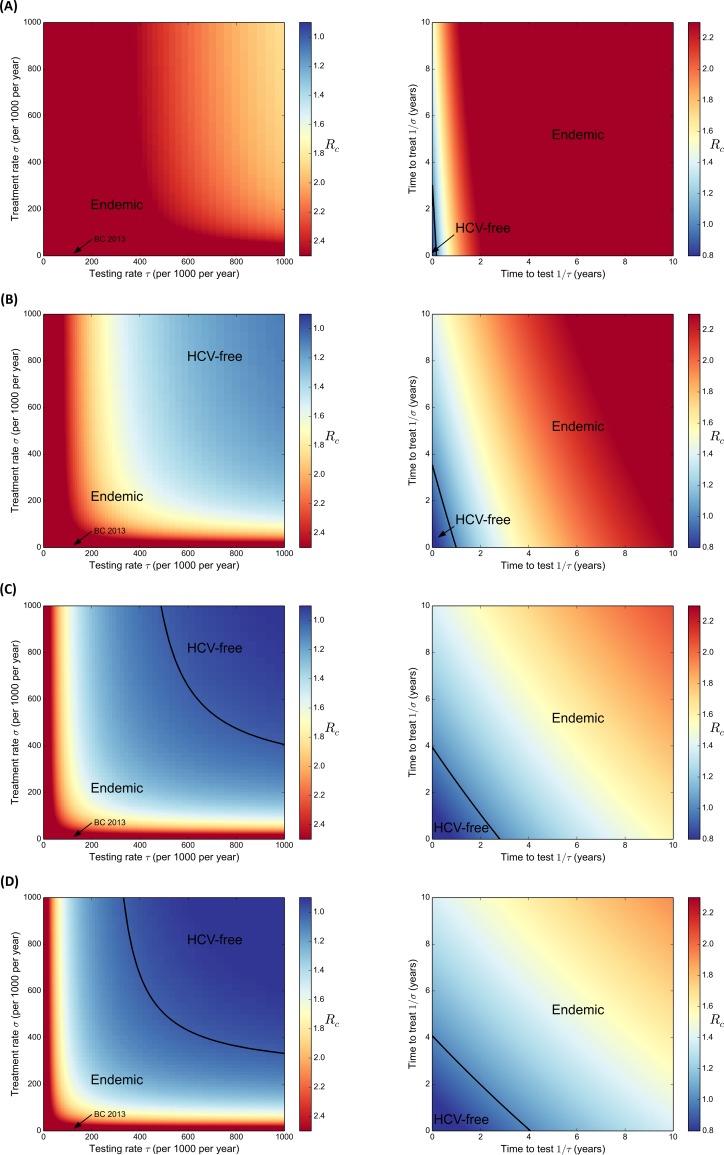
Estimated Control Reproduction Number for different HCV antiviral regimens: (A) Peg-IFN + RBV; (B) Peg-IFN + RBV plus (BOC or TPV); (C) IFN-free DAA; and (D) Best Case Scenario. In all scenarios, we assumed that: i) PWID moved into the risk reduction compartment after achieving SVR at a rate of 450 per 1000 PWID per year; and ii) PWID were eligible for treatment regardless of fibrosis level (i.e. *F*_0_ and higher).

Results of the *R*_*c*_ analysis are shown in the heat map plots (Fig 4). Colors indicate the magnitude of the *R*_*c*_ at that particular pair of values for testing and treatment rates; dark blue is the lowest and dark red is the highest. The black curve in each plot delimits the threshold at which *R*_*c*_ is equal to one, the area below the curve corresponds to *R*_*c*_ < 1, and the area above this curve corresponds to *R*_*c*_ > 1.

Based on preliminary results, we observed that if we calculate *R*_*c*_ considering the current treatment eligibility in BC (i.e. fibrosis level *F*_2_ and higher), we would never stabilize the epidemic and disease elimination would not be possible. As such, in this set of results, we considered a broad treatment eligibility in which PWID are treated regardless of their fibrosis level (i.e. fibrosis level *F*_0_ and higher). Thus, Fig 4 on the left-hand side (LHS) shows the estimates for *R*_*c*_ for different testing and treatment rates and for different types of antiviral regimens. Fig 4 on the right-hand side (RHS) corresponds to the same data now in terms of the time to testing (after the acute phase) and the time to access treatment after diagnosis. The figures on the RHS help us answer two questions: how often do people need to be tested and how soon after testing positive should they start treatment to achieve *R*_*c*_ < 1? Considering the Peg-IFN+RBV regimen, the model estimated that in BC, at the current testing and treatment rates, i.e. 140 and 8 per 1000 infected PWID per year, respectively, the *R*_*c*_ was 2.93 (Fig 4A LHS). In this scenario, based on an antiviral regimen with very low efficacy, there is very little opportunity to increase testing and treatment rates to obtain a *R*_*c*_ < 1, and PWID in this scenario would need to be tested and initiate treatment in a very short time after HCV infection (Fig 4A RHS). If the regimen was changed to Peg-IFN + RBV plus (BOC or TPV) the *R*_*c*_ was estimated to be 2.89 (Fig 4B LHS). In this scenario to obtain a *R*_*c*_ < 1, for example, if most PWID were tested within six months after the end of the acute phase, they would need to be treated within one year after the positive diagnosis (Fig 4B RHS). If the regimen was based on IFN-free DAA the estimated *R*_*c*_ was 2.72 (Fig 4C LHS) and in our ‘Best Case’ scenario the estimated *R*_*c*_ was 2.65 (Fig 4D LHS). Note that in these last two scenarios, we would need to drastically and efficiently increase testing and treatment rates to achieve disease elimination.

### Effect of Harm-Reduction

**Fig 5:**
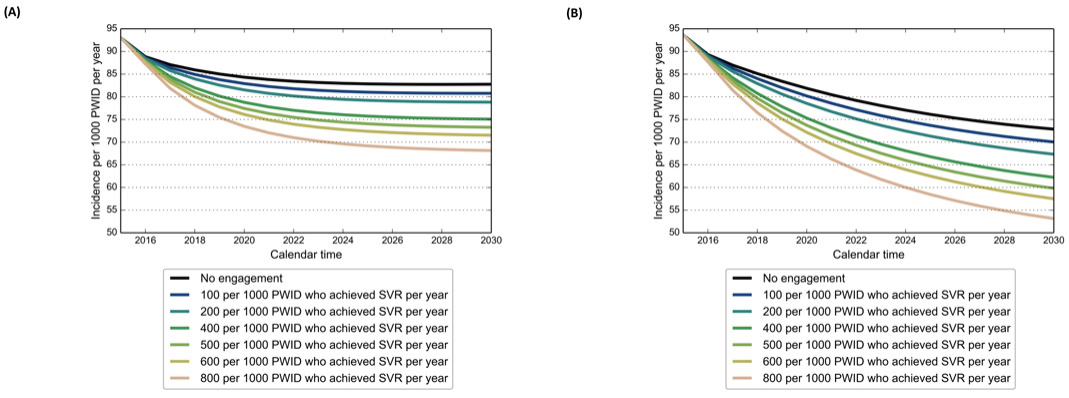
Model results for the estimated incidence rate from 2016 to 2030 for various scenarios of increased access to harm-reduction activities for PWID after achieving sustained virologic response; (A) Based on the current standard of care in British Columbia for HCV treatment eligibility (i.e. fibrosis level *F*_2_ and higher); (B) Based on a broad treatment eligibility in which PWID are treated regardless of their fibrosis level (i.e. fibrosis level *F*_0_ and higher). In all scenarios individuals received IFN-free DAAs. The ‘No engagement’ scenario only includes the background harm-reduction rate as per the Status Quo scenario. The other scenarios in these figures refer to the parameter *κ*, the rate in which individuals move into the Risk Reduction compartment (*R*). This intervention started to be implemented in 2016.

In Fig 5, we estimated the incidence rates from 2016 to 2030 for varying rates of increased access to harm-reduction activities for PWID who achieved SVR (parameter *κ*), while assuming a constant testing rate of 140 per 1000 infected PWID per year and a treatment rate of 80 per 1000 infected PWID per year using the IFN-free DAA regimen. Note that this intervention started to be implemented in the model in the year 2016 onward.

Fig 5(A) describes the effect of this intervention considering the current standard of care in BC for HCV treatment eligibility (i.e. fibrosis level *F*_2_ or higher), and Fig 5(B) describes this effect for a broad treatment eligibility in which PWID are treated regardless of their fibrosis level (i.e. fibrosis level *F*_0_ or higher). Thus, based on Fig 5(A), when we only have the background harm-reduction coverage as in the Status Quo scenario (black line), the estimated HCV incidence rate was 82.8 per 1000 PWID at the end of 2030. If, in addition to the background harm-reduction coverage, we also increased *κ* from 100 to 800 PWID per year (color lines), the incidence rate at the end of 2030 further declined to 80.8 for *κ* = 100 (2.4% decrease) or 68.1 for *κ* = 800 (17.7% decrease) per 1000 PWID per year.

If the treatment eligibility criterion was relaxed (Fig 5(B)), we obtained a much stronger effect of this intervention. Based on the background harm-reduction coverage as in the Status Quo scenario (black line), the estimated HCV incidence rate was 72.8 per 1000 PWID at the end of 2030. If, in addition to the background harm-reduction coverage, we also increased *κ* from 100 to 800 PWID per year (color lines), the incidence rate at the end of 2030 further declined to 70.0 for *κ* = 100 (3.9% decrease) or 53.1 for *κ* = 800 (27.1% decrease) per 1000 PWID.

### Sensitivity analysis

**Fig 6:**
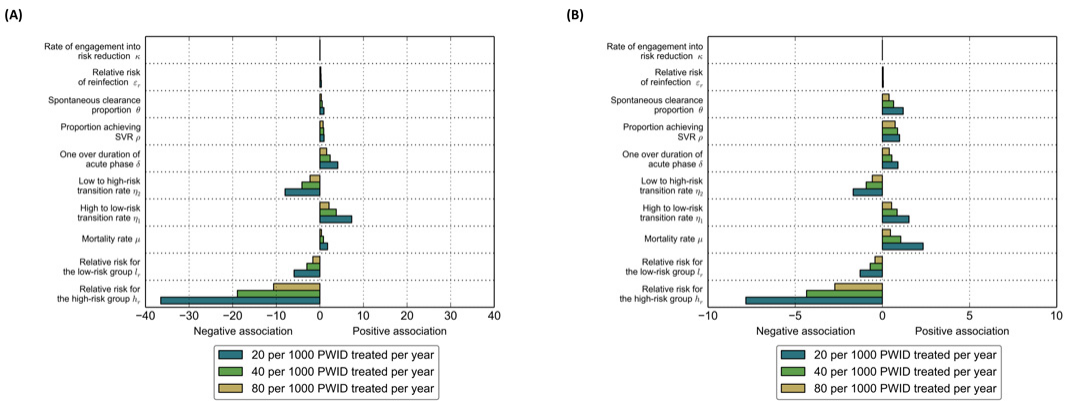
Results for the sensitivity analyses for the top ten parameters with the highest sensitivity coefficients: (A) HCV incidence rate at the end of 2030; and (B) HCV prevalence at the end of 2030.

We first estimated the univariate sensitivity coefficients for the HCV incidence rate (Fig 6(A)) and HCV prevalence (Fig 6(B)) changes under the three treatment uptake scenarios (i.e. increase treatment rate from 8 to 20, 40 and 80 per 1000 PWID per year) for the top ten parameters. Based on these two figures, we note the heterogeneity on the effect of each of these parameters on the estimated HCV incidence rate and prevalence. Overall, the most sensitive parameter was the relative risk for the high-risk group (*h*_*r*_), which shows that higher values for this parameter were associated with smaller decreases in HCV incidence rate and prevalence. Also note that the importance of this parameter decreased by increasing the treatment coverage rate in the population.

**Fig 7:**
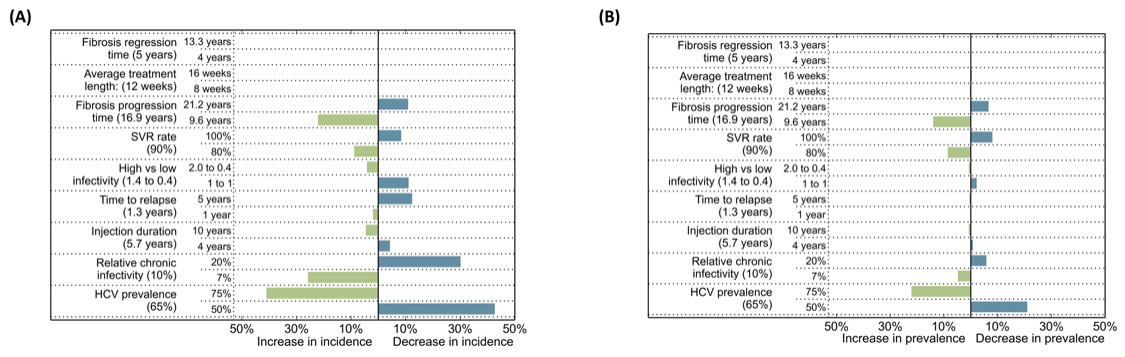
Results for the sensitivity analysis for the parameters with most uncertainty based on the available literature: (A) Percent change in HCV incidence in comparison to the Status Quo at the end of 2030; and (B) Percent change in HCV prevalence in comparison to the Status Quo at the end of 2030.

Secondly, we estimated the percent change in HCV incidence rate (Fig 7(A)) and HCV prevalence (Fig 7(B)), with respect to our model predictions based on the Status Quo scenario, for the parameters with the most uncertainty based on the available literature. The value for the HCV prevalence in BC in 2012 (i.e. Status Quo scenario) was the assumption that mostly influenced the changes in HCV incidence rate and prevalence at the end of 2030. If the BC HCV prevalence in the Status Quo scenario increased from 65% to 75%, we would expect that the HCV incidence rate would increase by approximately 43% and HCV prevalence would increase by approximately 21% at the end of 2030. If the BC HCV prevalence in the Status Quo scenario decreased from 65% to 50%, we would expect that the HCV incidence rate would decrease by approximately 41% and HCV prevalence would decrease by approximately 22% at the end of 2030. The two other assumptions that influenced the HCV incidence rate (although to a lesser extent) were the relative infectivity while chronically infected with respect to the acutely infected stage and the fibrosis progression time. For the HCV prevalence, the two other assumptions that influenced this outcome (also to a lesser extent) were the SVR rate and fibrosis progression time.

## Discussion

In this paper we analyzed the results of a mathematical model of HCV transmission, focused on PWID, developed to assess the impact of increasing HCV testing, treatment and engagement into harm-reduction activities, focused on active PWID, on the HCV epidemic in BC. Our results indicated that increased treatment coverage based on IFN-free DAA regimens, even at moderate levels, is a powerful strategy to decrease HCV incidence, prevalence and mortality. Furthermore, the impact of this intervention, coupled with an enhanced harm-reduction program focused on individuals highly vulnerable to re-infection after successfully completing therapy, can further decrease the impact of the HCV epidemic. This model has important public health and economic implications since it was commissioned by the BC Minister of Health, performed by the BC Centre for Excellence in HIV/AIDS and the BC Centre for Disease Control, to provide theoretical evidence and help inform on the feasibility of the proposed interventions and their potential impact on the burden associated with the HCV epidemic. In addition, this model sets the stage for a future cost-effectiveness analysis.

The combination of increased coverage of HCV treatment and access to different harm-reduction programs can be implemented in settings with heterogeneity in injecting practices in the population. This can be asserted since in a recent paper focused on a large cohort of active PWID in BC followed since 1996, the Vancouver Injection Drug Users Study, we demonstrated that cocaine and heroin injection have continuously been the preferred injecting drugs in BC and highly predictive of HCV infection [37]. Additionally, in this cohort, since 2005, the use of methamphetamine has been increasing, but is still at a lower level than the latter two illicit drugs. Thus, by considering in our harm-reduction component the combined efficacies of accessing needle exchange, drug treatment programs (pharmacological or not) and supervised injection sites, these interventions can be applied to a variety of drug use patterns in a population. However, some may argue that these interventions will not work for some PWID at the highest risk of HCV transmission because these individuals may be the most difficult ones to engage and to retain in treatment and in harm-reduction programs. Therefore, to maximize the public health impact of these interventions, we will need an optimized implementation strategy, which particularly emphasizes the need to reach, support, treat and protect these individuals [8].

One critical aspect for the success of the proposed interventions is to identify and test individuals unaware that they are HCV-positive [38, 39]. To do so, it is important that different agencies in our healthcare system implement an aggressive “seek” campaign to identify and test these individuals. Testing should focus more on HCV RNA testing as this actively tests for virus as opposed to HCV antibody testing which only tests for previous exposure. Currently, testing often occurs in relation to risk-based testing or for clinical diagnosis of late-stage liver disease. In addition, the determination of liver fibrosis should focus on cheaper, safer and less-invasive methodology (e.g., Fibroscan) as opposed to liver biopsy, which has been the gold-standard for many years [40]. As many PWID also suffer from mental illnesses or other comorbid conditions and are often current injectors, there is some reluctance on the part of healthcare providers to engage these individuals into HCV assessment and treatment due to fears of non-adherence to the antiviral regimens. However, there are now international recommendations that PWID should be offered HCV treatment based on data that SVR is similar among PWID and non-PWID [41]. With new and better-tolerated antivirals on the horizon, such as IFN-free DAAs, adherence could be addressed by directly observed therapy, consistent with the approach taken to treat opioid dependence in BC (i.e. daily witnessed ingestion of medication at a registers’ pharmacy). However, an aggressive seek, test and treat approach would still be necessary as would a retention in care and harm-reduction strategy to minimize the risk of re-infection. The individual and public health consequences of treating these individuals at a late stage of their disease are enormous, since their quality of life and survival have been significantly reduced, and they may incur substantial costs to the healthcare system due to care requirements linked to end-stage liver disease [42, 43].

This model is unique in different aspects and it has important implications. First, we included movement across different fibrosis states in this population to inform treatment eligibility and success. Knowing the distribution of fibrosis states is known to currently have a direct effect in determining treatment eligibility in many jurisdictions and efficacy, to affect the progression of liver disease, and to be associated with both direct medical costs and health-related quality of life [35]. Given the high uncertainty in this parameter, based on the sensitivity analysis, we showed that both changes in incidence and prevalence at 15 years were not significantly influenced by the values used for these transition rates. In future versions of this model, in view of new data on disease progression based on IFN-free DAAs through the different ongoing clinical trials, we plan to fine-tune this model, specifically focusing on the effect of treatment on the distribution of fibrosis states in a population. Second, this model provides an independent validation of the results in *Martin et al*. supporting the notion that HCV treatment can be used to reduce the HCV incidence, prevalence and mortality among PWID [21, 22]. It is important to mention that this model is quite different from the seminal paper by *Martin et al*. in 2013 assessing the impact of treatment on the HCV epidemic among PWID using INF-free DAA in several aspects, including [22]: (1) it used data from a community-based cohort study conducted in Vancouver (N = 2913) instead of being based on the overall BC population infected or at risk of being infected with HCV; (2) it did not model the effect of fibrosis progression or changes in treatment eligibility on the projected course of the HCV epidemic; and (3) it did not model changes in access to harm-reduction programs which have been shown to further decrease the population impact of this epidemic. Third, in this model we showed the synergistic effect of using the strategies “HCV Treatment as Prevention” and increased harm-reduction coverage on the HCV epidemic among PWID. In this case, the harm-reduction component played two roles: to prevent premature mortality and to reduce the risk of onward transmission. Thus, the effect of this strategy on the estimated incidence rate is probably due to the lower risk of reinfection among PWID. Fourth, through our different cohort studies of PWID in Vancouver we are in a favorable position to test the interventions of this study since the PWID population in these cohorts is quite heterogeneous (e.g., they include young and old PWID, high and low risk PWID, individuals with mental health issues and HIV/HCV co-infected), thus allowing us to assess the feasibility of these interventions in a variety of risk groups within the PWID population. At last, the knowledge gained from the proposed interventions tested through this model can offer insights into the development and implementation of new prevention strategies, inform policy and decision makers, and suggest possible directions for future investigation.

This model also has some limitations. First, we assumed a homogenous mixing in the PWID population, thus simplifying the complexities that exist in the sharing networks of these individuals. Second, the sensitivity analysis showed that the estimated HCV prevalence and incidence were most sensitive to the average contact rate that incorporates, among several parameters, the HCV transmission probability. Based on different studies, we can divide the HCV natural history in two phases with distinct infectivity based on the HCV RNA levels [5, 44, 45]. Unfortunately, there is no definite evidence on what the associated transmission probabilities for these phases are. Third, although the mortality rates associated with liver disease, among PWID infected with HCV in BC, were high, the literature has several studies with mortality data amongst PWID consistent with the one used in this study [46–48]. Finally, we did not model the impact of the proposed interventions comparing the HCV mono-infected population and the individuals co-infected with HCV and HIV. In the PWID HIV-positive population, HCV prevalence is estimated as high as 90%, and HCV progression is much faster [49]. Thus, in the future, we plan extend the present model to specifically investigate the impact of the proposed interventions on the HIV/HCV co-infected PWID population.

In conclusion, we provided theoretical support that the new IFN-free DAAs have the potential to cure more individuals, and their impact on the HCV burden in BC’s PWID population can be strongly enhanced by effective evidence-based initiatives that identify, treat and engage individuals into long-term risk reduction. However, unless we increase the current levels of treatment and testing, the HCV epidemic among PWID in BC, and in other parts of the world with similar epidemiological background, will remain a substantial public health concern for many years.

## Supporting Information

S1 File Differential Equations, Parameters, Initial Conditions and Data Sources. (PDF)
