# Supplementary material for: Are Interferon-Free Direct-Acting Antivirals for the Treatment of HCV Enough to Control the Epidemic among People Who Inject Drugs?
Source: PLoS One. 2015 Dec 3;10(12):e0143836. doi: 10.1371/journal.pone.0143836 (PMC4669174; doi:10.1371/journal.pone.0143836)
Supplement: S1 File — (PDF) [file pone.0143836.s001.pdf]

Supporting Information

Title: Are interferon-free direct-acting antivirals for the treatment of HCV enough to control the epidemic among people who inject drugs?

Authors: Viviane D. Lima*, Ignacio Rozada, Jason Grebely, Mark Hull, Lillian Lourenco, Bohdan Nosyk, Mel Krajden, Eric Yoshida, Evan Wood, Julio S.G. Montaner

*To whom correspondence should be addressed. E-mail:vlima@cfenet.ubc.ca

Pages	Section
**2 – 4**	**Differential Equations**
**5 – 15**	**Table 1. Model Parameters and Initial Conditions**
**16**	**Detailed analysis of the Results for Figure 3 in the manuscript**
**17 – 20**	**References**

Differential Equations

The full model has 105 differential equations (i.e. seven primary health states with five fibrosis stage sub-compartments, and further divided into three injection risk categories), and for simplicity in writing these equations, we used a vector notation in which each vector, one for each compartment, encompasses its five sub-components, one for each fibrosis stage (e.g., $\vec{S}=\left[S_0,S_1,S_2,S_3,S_4\right]^T$).

The explanation and assumptions for all parameters can be found in Supporting Information Table 1. The differential equations for each of the three risk categories (with *i* = 0,1,2 corresponding to zero, low and high injection risk, respectively) are given by:

Susceptible compartments

$$\frac{d\vec{S}_2}{dt}=n_2\Pi+\rho\left(1-\kappa\right)\Omega\vec{T}_2+\gamma\vec{R}_2+\theta\delta\vec{I}_2+\psi\vec{S}_2+c_1\vec{S}_0+c_3\vec{S}_1-\left(\lambda_{S_2}+\mu+c_0+c_2\right)\vec{S}_2$$

$$\frac{d\vec{S}_1}{dt}=n_1\Pi+\rho\left(1-\kappa\right)\Omega\vec{T}_1+\gamma\vec{R}_1+\theta\delta\vec{I}_1+\psi\vec{S}_1+c_1\vec{S}_0+c_2\vec{S}_2-\left(\lambda_{S_1}+\mu+c_0+c_3\right)\vec{S}_1$$

$$\frac{d\vec{S}_0}{dt}=\theta\delta\vec{I}_0+\psi\vec{S}_0+c_0\left(\vec{S}_1+\vec{S}_2\right)-\left(\mu+2c_1\right)\vec{S}_0$$

Acutely infected compartments

$$\frac{d\vec{I}_2}{dt}=\lambda_{S_i}\vec{S}_2+\lambda_{R_i}\vec{R}_2+c_1\vec{I}_0+c_3\vec{I}_1-\left(\delta+F+\mu+c_0+c_2\right)\vec{I}_2$$

$$\frac{d\vec{I}_1}{dt}=\lambda_{S_1}\vec{S}_1+\lambda_{R_1}\vec{R}_1+c_1\vec{I}_0+c_2\vec{I}_2-\left(\delta+F+\mu+c_0+c_3\right)\vec{I}_2$$

$$\frac{d\vec{I}_0}{dt}=c_0\left(\vec{I}_1+\vec{I}_2\right)-\left(\delta+F+\mu+2c_1\right)\vec{I}_0$$

Chronic unaware compartments

$$\frac{d\vec{C}_{u2}}{dt}=\left(1-\theta\right)\delta\vec{I}_2+c_1\vec{C}_{u0}+c_3\vec{C}_{u1}-\left(\tau+F+\mu+c_0+c_2\right)\vec{C}_{u2}$$

$$\frac{d\vec{C}_{u1}}{dt}=\left(1-\theta\right)\delta\vec{I}_1+c_1\vec{C}_{u0}+c_2\vec{C}_{u2}-\left(\tau+F+\mu+c_0+c_3\right)\vec{C}_{u1}$$

$$\frac{d\vec{C}_{u0}}{dt}=\left(1-\theta\right)\delta\vec{I}_0+c_0\left(\vec{C}_{u1}+\vec{C}_{u2}\right)-\left(\tau+F+\mu+2c_1\right)\vec{C}_{u0}$$

Chronic aware and eligible for treatment compartments

$$\frac{d\vec{C}_{a2}}{dt}=\tau\vec{C}_{u2}+\left(1-\rho\right)\omega\vec{T}_2+c_1\vec{C}_{a0}+c_3\vec{C}_{a1}-\left(\sigma_{C_a}+F+\mu+c_0+c_2\right)\vec{C}_{a2}$$

$$\frac{d\vec{C}_{a1}}{dt}=\tau\vec{C}_{u1}+\left(1-\rho\right)\omega\vec{T}_1+c_1\vec{C}_{a0}+c_2\vec{C}_{a2}-\left(\sigma_{C_a}+F+\mu+c_0+c_3\right)\vec{C}_{a1}$$

$$\frac{d\vec{C}_{a0}}{dt}=\tau\vec{C}_{u0}+\left(1-\rho\right)\omega\vec{T}_0+c_0\left(\vec{C}_{a1}+\vec{C}_{a2}\right)-\left(\sigma_{C_a}+F+\mu+2c_1\right)\vec{C}_{a0}$$

Chronic aware and not eligible for treatment compartments

$$\frac{d\vec{C}_{n2}}{dt}=\zeta\sigma_{C_a}\vec{C}_{a2}+c_1\vec{C}_{n0}+c_3\vec{C}_{n1}-\left(\sigma_{C_n}+F+\mu+c_0+c_2\right)\vec{C}_{n2}$$

$$\frac{d\vec{C}_{n1}}{dt}=\zeta\sigma_{C_a}\vec{C}_{a1}+c_1\vec{C}_{n0}+c_2\vec{C}_{n2}-\left(\sigma_{C_n}+F+\mu+c_0+c_3\right)\vec{C}_{n1}$$

$$\frac{d\vec{C}_{n0}}{dt}=\zeta\sigma_{C_a}\vec{C}_{a0}+c_0\left(\vec{C}_{n1}+\vec{C}_{n2}\right)-\left(\sigma_{C_n}+F+\mu+2c_1\right)\vec{C}_{n0}$$

On treatment compartments

$$\frac{d\vec{T}_2}{dt}=\left(1-\zeta\right)\sigma_{C_a}\vec{C}_{a2}+\sigma_{C_n}\vec{C}_{n2}+c_1\vec{T}_0+c_3\vec{T}_1-\left(\omega+\mu+c_0+c_2\right)\vec{T}_2$$

$$\frac{d\vec{T}_1}{dt}=\left(1-\zeta\right)\sigma_{C_a}\vec{C}_{a1}+\sigma_{C_n}\vec{C}_{n1}+c_1\vec{T}_0+c_2\vec{T}_2-\left(\omega+\mu+c_0+c_3\right)\vec{T}_1$$

$$\frac{d\vec{T}_0}{dt}=\left(1-\zeta\right)\sigma_{C_a}\vec{C}_{a0}+\sigma_{C_n}\vec{C}_{n0}+c_0\left(\vec{T}_1+\vec{T}_2\right)-\left(\omega+\mu+2c_1\right)\vec{T}_0$$

Risk reduction compartments

$$\frac{d\vec{R}_2}{dt}=\rho\kappa\Omega\vec{T}_2+\Psi\vec{R}_2+c_1\vec{R}_0+c_3\vec{R}_1-\left(\gamma+\lambda_{R_2}+\mu+c_0+c_2\right)\vec{R}_2$$

$$\frac{d\vec{R}_1}{dt}=\rho\kappa\Omega\vec{T}_1+\Psi\vec{R}_1+c_1\vec{R}_0+c_2\vec{R}_2-\left(\gamma+\lambda_{R_1}+\mu+c_0+c_3\right)\vec{R}_1$$

$$\frac{d\vec{R}_0}{dt}=\rho\Omega\vec{T}_i+\Psi\vec{R}_0+c_0\left(\vec{R}_1+\vec{R}_2\right)-\left(\mu+2c_1\right)\vec{R}_i$$

Table 1. Model Parameters and Initial Conditions

**Parameter**	**Explanation and Values**	**References**
$\vec{S}=\left[S_0,S_1,S_2,S_3,S_4\right]^T$	Vector for Susceptible individuals: its components correspond to the five fibrosis levels. Initial values are for stable endemic equilibrium. The definition of this can be done in terms of the Control Reproduction Number (R_c). A $R_c<1$ means that, over time, the model will reach the disease-free equilibrium - meaning that on average an infected individual produces less than one new infection over the course of his/her infectious period, and thus, the epidemic cannot grow. A $R_c>1$ means that, over time, the model will reach a stable endemic equilibrium - meaning that each infected individual produces, on average, more than one new infection, and the disease continues to spread in the population.	
$\vec{I}=\left[I_0,I_1,I_2,I_3,I_4\right]^T$	Vector for Acutely Infected individuals: its components correspond to the five fibrosis levels. Initial values are for stable endemic equilibrium.	
$\vec{C}_u=\left[C_{u_0},C_{u_1},C_{u_2},C_{u_3},C_{u_4}\right]^T$	Vector for Chronic unaware individuals: its components correspond to the five fibrosis levels.	

	Initial values are for stable endemic equilibrium.	
$\vec{C}_a = \left[C_{a_0}, C_{a_1}, C_{a_2}, C_{a_3}, C_{a_4}\right]^T$	Vector for Chronic aware individuals: its components correspond to the five fibrosis levels. Initial values are for stable endemic equilibrium.	
$\vec{C}_n = \left[C_{n_0}, C_{n_1}, C_{n_2}, C_{n_3}, C_{n_4}\right]^T$	Vector for individuals Chronic aware and not eligible for treatment: its components correspond to the five fibrosis levels. Initial values are for stable endemic equilibrium.	
$\vec{T} = \left[T_0, T_1, T_2, T_3, T_4\right]^T$	Vector for individuals on Treatment: its components correspond to the five fibrosis levels. Initial values are for stable endemic equilibrium.	
$\vec{R} = \left[R_0, R_1, R_2, R_3, R_4\right]^T$	Vector for individuals on risk reduction: its components correspond to the five fibrosis levels. Initial values are for stable endemic equilibrium.	
ρ	Proportion on Peg-IFN + RBV achieving SVR: 55%;<br>Proportion on Peg-IFN + RBV plus (BOC or TPV) achieving SVR: 70%;<br>Proportion on IFN-free DAA achieving SVR: 90%;<br>Proportion on the Best Case Scenario achieving SVR: 100%.	[1-7]
κ	Rate of PWID moving into risk reduction after achieving SVR:<br>Baseline scenario: 0 per 1000 PWID per year;	

	Intervention scenarios: 100, 200, 400, 500, 600 and 800 per 1000 PWID per year.	
Ω	Transition matrix across fibrosis stages after achieving SVR given by: $\Omega = \begin{pmatrix} 1 & 1 & 0 & 0 & 0 \\ 0 & 0 & 1 & 0 & 0 \\ 0 & 0 & 0 & 1 & 0 \\ 0 & 0 & 0 & 0 & 1 \\ 0 & 0 & 0 & 0 & 0 \end{pmatrix}$ We assume that PWID who achieve SVR improve their fibrosis by one level. Thus, based on this matrix, PWID in T_4 can transition to either R_3 or S_3; PWID in T_3 can transition to either R_2 or S_2; PWID in T_2 can transition to either R_1 or S_1; and PWID in T_1 and T_0 can transition to either R_0 or S_0 after achieving SVR.	
γ	Loss of individuals from the risk reduction compartment: 60 per 1000 PWID per year	[8]
θ	Spontaneous clearance proportion: 25%.	[9]
$1/\delta$	Duration of acute phase: 0.5 years.	[7, 10]
Ψ	Fibrosis regression rate matrix:	

	$$\Psi=\begin{pmatrix} 0 & \psi & 0 & 0 & 0 \\ 0 & -\psi & \psi & 0 & 0 \\ 0 & 0 & -\psi & \psi & 0 \\ 0 & 0 & 0 & -\psi & \psi \\ 0 & 0 & 0 & 0 & -\psi \end{pmatrix}$$ PWID who achieved SVR gradually improve their liver condition, which translates into decreases in their fibrosis level. In the model, PWID exit compartments S_i and R_i at a yearly rate of ψ, and arrive at compartments S_{i-1} and R_{i-1} with the same rate ψ, respectively, for $i=1$ to 4.	
ψ	Fibrosis regression rate from F_j to F_{j-1} for $j=1,2,3,4$: 0.2 per year	[11, 12]
F	Fibrosis progression rate matrix: $$F=\begin{pmatrix} f_{01} & 0 & 0 & 0 & 0 \\ -f_{01} & f_{12} & 0 & 0 & 0 \\ 0 & -f_{12} & f_{23} & 0 & 0 \\ 0 & 0 & -f_{23} & f_{34} & 0 \\ 0 & 0 & 0 & -f_{34} & 0 \end{pmatrix}$$ HCV infected individuals gradually deteriorate their liver condition, which translates into increases in their fibrosis level. In the model, individuals exit compartments $I_i, C_{u_i}, C_{a_i}, C_{n_i}$ at a yearly rate of $f_{i,i+1}$, and arrive at compartment $I_{i+1}, C_{u_{i+1}}, C_{a_{i+1}}, C_{n_{i+1}}$ with	

	the same rate $f_{i,i+1}$, respectively, for $i = 0$ to 3.	
$\left[f_{01}, f_{12}, f_{23}, f_{34}\right]$	Baseline transition rates: f_{01} = 0.067 per year; f_{12} = 0.049 per year; f_{23} = 0.069 per year; f_{34} = 0.051 per year. These parameters were determined from a weighted average of sex and age-specific transition rates based on the paper by *Razavi et al.* [13]. The choice of age group was based on the top seven age groups in which the HCV incidence was the highest in the *Remis report*, i.e., 15-49 years [14]. A sensitivity analysis was performed using data from a meta-analysis by *Thein et al.* [15], which has faster transition rates f_{01} = 0.109 per year; f_{12} = 0.068 per year; f_{23} = 0.113 per year; f_{34} = 0.125 per year. Another sensitivity analysis was performed using data from *Razavi et al.* [13], which also had values for slower transition rates f_{01} = 0.054 per year; f_{12} = 0.039 per year; f_{23} = 0.051 per year; f_{34} = 0.041 per year.	[13-15]
τ	Status Quo testing rate: 140 per 1000 PWID per year; The only intervention for this parameter was to assume immediate testing at the end of the acute	[16, 17]

	phase.	
$1/\omega$	Duration of Peg-IFN + RBV treatment: 48 weeks depending on the genotype;<br>Duration of Peg-IFN + RBV plus (BOC or TPV) treatment: 24 to 48 weeks depending on the genotype;<br>Duration of IFN-free DAA treatment: 12 weeks regardless of genotype;<br>Duration of the Best Case Scenario treatment: 1 week regardless of genotype.	[2, 4, 6, 7, 18-21]
σ_{C_a}	Status Quo treatment rate for individuals in the C_a compartment: 8 per 1000 PWID per year;<br>Intervention scenarios for the treatment rate for individuals in the C_a compartment: 20, 40 and 80 per 1000 PWID per year	[16, 17]
σ_{C_n}	Treatment rate for individuals in the C_n compartment: $\alpha\sigma_{C_a}$	
α	Reduction in the treatment rate for C_n due to treatment ineligibility:<br>Based on Peg-IFN + RBV treatment: 0.10;<br>Based on Peg-IFN + RBV plus (BOC or TPV) treatment: 0.20;	[4, 16, 22]

	Based on IFN-free DAA treatment: 0.75;<br>Based on Best Case Scenario treatment: 1.00.<br>These numbers were derived based on the unstable housing and drug use patterns in these reports, counter-indications for the use of each of these drugs and the Canadian HCV treatment guidelines.	
ζ	Proportion of PWID not eligible for treatment:<br>Based on Peg-IFN + RBV treatment: 90%;<br>Based on Peg-IFN + RBV plus (BOC or TPV) treatment: 75%. This is probably optimistic due to the fact that BOC/TPV are not very well tolerated [23];<br>Based on IFN-free DAA treatment: 20%. We have no data on this other than phase 3 reports.<br>Based on Best Case Scenario treatment: 0%.	[14, 17, 23]
Π	Recruitment rate into susceptible PWID. For lack of better data, this constant was fit in the model to maintain a constant population. It was estimated using: $\sum_{j=0}^{2}\left(\mu\cdot\left(S_j+I_j+C_{u_j}+C_{a_j}+C_{n_j}+T_j+R_j\right)\right)$, with j representing the 3 risk categories (i.e., zero, low and high risk).	
λ_{S_i}	Force of infection for the high and low risk categories:	

	$\lambda_{S_k} = h_k \beta \sum_{i=1}^{i=2} \left(h_i \sum_{j=0}^{4} \left(I_{ij} + \chi_0 \left(C_{u_{ij}} + C_{a_{ij}} + C_{n_{ij}} \right) + \chi_1 T_{ij} \right) \right) \Big/ N$<br><br>, with k=1 (low risk), 2 (high risk), *i*= 1 (low risk), 2 (high risk), and *j*=0,…, 4 fibrosis level<br>Force of infection for the zero-risk category: $\lambda_{S_0} = 0$	
β	Average contact rate. This parameter was numerically derived to fit a fixed prevalence. In BC, it is estimated that 65% of the PWID population is HCV RNA positive. With all other parameters fixed, we ran a long-time simulation inside a Nelder-Mead simplex algorithm that determined the optimal value of β that results in a 65% chronic prevalence in the endemic scenario. Note that this parameter includes the background rate of individuals in the population currently accessing harm-reduction programs (53 per 100 PWID per year)	[22, 24]
χ_0	Relative infectivity while chronically infected with respect to the acutely infected stage: 10%. This parameter is to take into account the different phases in the HCV life cycle and their respective infectivity. Thus in this case, the infectivity in the acute infected stage was assumed to be 100%. This means that in	[10, 25, 26]

	the very short period of time during the acute phase individuals are very infectious and once they achieve the chronic stage they are much less infectious, although they remain infectious for a long period of time.	
χ_1	Relative infectivity while on treatment with respect to the acutely infected stage: 5%.	[10, 25, 26]
N	Total PWID population was 18,068 and given by $\sum_{i=1}^{2}\sum_{j=0}^{4}\left(S_{ij}+I_{ij}+C_{u_{ij}}+C_{a_{ij}}+C_{n_{ij}}+T_{ij}+R_{ij}\right)$, *i*= 1 (low risk), 2 (high risk), *j*=0,…, 4 fibrosis level. This was derived from the overall HCV prevalence. The total population including the zero risk category was fit in order for N to match the high and low risk compartments	
h_2	Increased relative risk for high-risk individuals: 1.40	[27]
h_1	Decreased relative risk for low-risk individuals: 0.40	[28]
n_2	Proportion of the PWID that engages in high risk injection drug use: 64%	[22, 24]
n_1	Proportion of the PWID that engages in low risk injection drug use: 36%	[22, 24]
λ_{R_i}	Force of re-infection was estimated using: $\left(1-\varepsilon_R\right)\lambda_{S_i}$	

ε_R	Re-infection risk reduction due to harm-reduction and natural causes: 79%	[28]
μ_{PWID}	Status Quo mortality rate for PWID with fibrosis level F_0: 0.01 per person-years	[14, 29]
m_{LD}	Standardized mortality ratio for liver-related disease: 8.44. This was determined from deaths attributable to viral hepatitis, liver cancer, and alcoholic and non-alcoholic liver disease in a population of HCV infected individuals	[30, 31]
μ	Population mortality rate range across fibrosis stages. These set of parameters were linearly interpolated between the baseline mortality rate (μ_{PWID}) and the SMR-adjusted value for individuals who died from liver-related disease ($m_{LD}\mu_{PWID}$)	
c_0	Cessation rate for the high and low-risk individuals: 0.18 per PWID per year	[32]
c_1	Relapse rate back to high-risk drug use: 0.75 per PWID per year	[32]
c_2	High to low-risk transition rate: 0.32 per PWID per year	[22, 24]
c_3	Low to high-risk transition rate. This rate was estimate to keep the PWID population constant at	

	64% having high risk and 36% having low risk.	

Abbreviations: PWID: people who inject drugs, Peg-IFN: pegylated interferon, RBV: ribavirin, BOC: boceprevir, TPV: telaprevir, SVR: sustained virologic response, DAA: direct-acting antivirals; SMR: standardized mortality ratio.

Statistical analysis for the trend in the distribution of fibrosis levels in Figure 3 in the manuscript

(A)

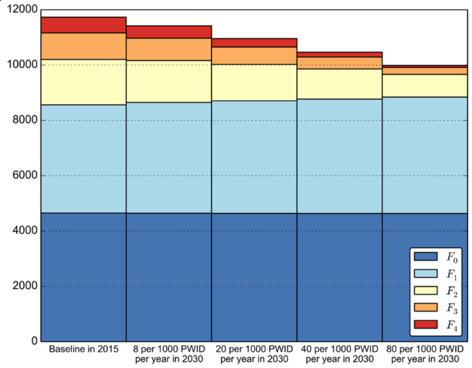

Fibrosis Level	Baseline in 2015	8 per 1000 PWID per year in 2030	20 per 1000 PWID per year in 2030	40 per 1000 PWID per year in 2030	80 per 1000 PWID per year in 2030	P-value for Trend
F0	4649 (40%)	4643 (41%)	4638 (42%)	4634 (44%)	4636 (46%)	<0.0001
F1	3912 (33%)	4010 (35%)	4067 (37%)	4132 (39%)	4204 (42%)	<0.0001
F2	1636 (14%)	1507 (13%)	1317 (12%)	1091 (10%)	824 (8%)	<0.0001
F3	959 (8%)	809 (7%)	626 (6%)	431 (4%)	248 (2%)	<0.0001
F4	573 (5%)	445 (4%)	308 (3%)	175 (2%)	71 (1%)	<0.0001
Total	11730 (100%)	11413 (100%)	10956 (100%)	10464 (100%)	9982 (100%)	

(B)

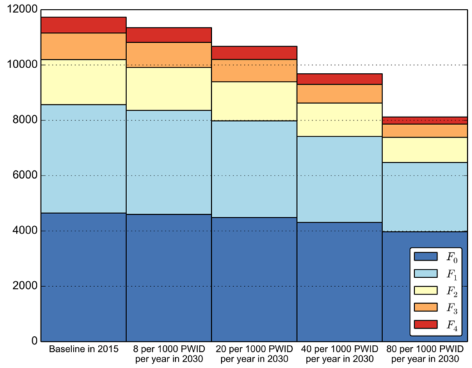

Fibrosis Level	Baseline in 2015	8 per 1000 PWID per year in 2030	20 per 1000 PWID per year in 2030	40 per 1000 PWID per year in 2030	80 per 1000 PWID per year in 2030	P-value for Trend
F0	4649 (40%)	4597 (41%)	4487 (42%)	4308 (44%)	3967 (49%)	<0.0001
F1	3912 (33%)	3760 (33%)	3492 (33%)	3106 (32%)	2508 (31%)	0.0001
F2	1636 (14%)	1554 (14%)	1412 (13%)	1210 (12%)	908 (11%)	<0.0001
F3	959 (8%)	903 (8%)	809 (8%)	676 (7%)	480 (6%)	<0.0001
F4	573 (5%)	535 (5%)	472 (4%)	383 (4%)	256 (3%)	<0.0001
Total	11730 (100%)	11348 (100%)	10672 (100%)	9682 (100%)	8119 (100%)	

(C)

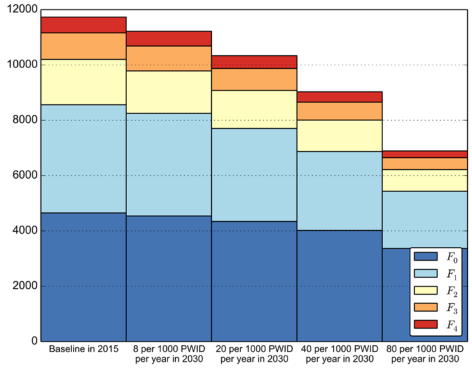

Fibrosis Level	Baseline in 2015	8 per 1000 PWID per year in 2030	20 per 1000 PWID per year in 2030	40 per 1000 PWID per year in 2030	80 per 1000 PWID per year in 2030	P-value for Trend
F0	4649 (40%)	4540 (40%)	4343 (42%)	4012 (44%)	3364 (49%)	<0.0001
F1	3912 (33%)	3706 (33%)	3362 (33%)	2860 (32%)	2071 (30%)	<0.0001
F2	1636 (14%)	1537 (14%)	1371 (13%)	1135 (13%)	778 (11%)	<0.0001
F3	959 (8%)	897 (8%)	794 (8%)	648 (7%)	432 (6%)	<0.0001
F4	573 (5%)	533 (5%)	467 (5%)	376 (4%)	243 (4%)	<0.0001
Total	11730 (100%)	11213 (100%)	10338 (100%)	9030 (100%)	6888 (100%)	

References

1. Dimova RB, Zeremski M, Jacobson IM, Hagan H, Des Jarlais DC, Talal AH. Determinants of hepatitis C virus treatment completion and efficacy in drug users assessed by meta-analysis. Clinical infectious diseases : an official publication of the Infectious Diseases Society of America. 2013;56(6):806-16. Epub 2012/12/12. doi: 10.1093/cid/cis1007. PubMed PMID: 23223596; PubMed Central PMCID: PMC3582354.

2. Feld JJ. Interferon-free strategies with a nucleoside/nucleotide analogue. Semin Liver Dis. 2014;34(1):37-46. Epub 2014/05/02. doi: 10.1055/s-0034-1371009. PubMed PMID: 24782257.

3. Hellard M, Sacks-Davis R, Gold J. Hepatitis C treatment for injection drug users: a review of the available evidence. Clinical infectious diseases : an official publication of the Infectious Diseases Society of America. 2009;49(4):561-73. Epub 2009/07/11. doi: 10.1086/600304. PubMed PMID: 19589081.

4. Hull M, Klein M, Shafran S, Tseng A, Giguere P, Cote P, et al. CIHR Canadian HIV Trials Network Coinfection and Concurrent Diseases Core: Canadian guidelines for management and treatment of HIV/hepatitis C coinfection in adults. Can J Infect Dis Med Microbiol. 2013;24(4):217-38. Epub 2014/02/04. PubMed PMID: 24489565; PubMed Central PMCID: PMC3905006.

5. Jennings CL, Sherman KE. Hepatitis C and HIV co-infection: new drugs in practice and in the pipeline. Curr HIV/AIDS Rep. 2012;9(3):231-7. Epub 2012/05/29. doi: 10.1007/s11904-012-0122-z. PubMed PMID: 22638982; PubMed Central PMCID: PMC3551247.

6. Lawitz E, Lalezari JP, Hassanein T, Kowdley KV, Poordad FF, Sheikh AM, et al. Sofosbuvir in combination with peginterferon alfa-2a and ribavirin for non-cirrhotic, treatment-naive patients with genotypes 1, 2, and 3 hepatitis C infection: a randomised, double-blind, phase 2 trial. Lancet Infect Dis. 2013;13(5):401-8. Epub 2013/03/19. doi: 10.1016/S1473-3099(13)70033-1. PubMed PMID: 23499158.

7. Rosen HR. Clinical practice. Chronic hepatitis C infection. N Engl J Med. 2011;364(25):2429-38. Epub 2011/06/24. doi: 10.1056/NEJMcp1006613. PubMed PMID: 21696309.

8. Dalgard O. Follow-up studies of treatment for hepatitis C virus infection among injection drug users. Clinical infectious diseases : an official publication of the Infectious Diseases Society

of America. 2005;40 Suppl 5:S336-8. Epub 2005/03/16. doi: 10.1086/427449. PubMed PMID: 15768344.

9. Grebely J, Page K, Sacks-Davis R, van der Loeff MS, Rice TM, Bruneau J, et al. The effects of female sex, viral genotype, and IL28B genotype on spontaneous clearance of acute hepatitis C virus infection. Hepatology. 2014;59(1):109-20. Epub 2013/08/03. doi: 10.1002/hep.26639. PubMed PMID: 23908124; PubMed Central PMCID: PMC3972017.

10. Mondelli MU, Cerino A, Cividini A. Acute hepatitis C: diagnosis and management. Journal of hepatology. 2005;42 Suppl(1):S108-14. Epub 2005/03/22. doi: 10.1016/j.jhep.2004.10.017. PubMed PMID: 15777565.

11. George SL, Bacon BR, Brunt EM, Mihindukulasuriya KL, Hoffmann J, Di Bisceglie AM. Clinical, virologic, histologic, and biochemical outcomes after successful HCV therapy: a 5-year follow-up of 150 patients. Hepatology. 2009;49(3):729-38. doi: 10.1002/hep.22694. PubMed PMID: 19072828; PubMed Central PMCID: PMC2731713.

12. Shiratori Y, Imazeki F, Moriyama M, Yano M, Arakawa Y, Yokosuka O, et al. Histologic improvement of fibrosis in patients with hepatitis C who have sustained response to interferon therapy. Annals of internal medicine. 2000;132(7):517-24. PubMed PMID: 10744587.

13. Razavi H, Waked I, Sarrazin C, Myers RP, Idilman R, Calinas F, et al. The present and future disease burden of hepatitis C virus (HCV) infection with today's treatment paradigm. J Viral Hepat. 2014;21 Suppl 1:34-59. Epub 2014/04/10. doi: 10.1111/jvh.12248. PubMed PMID: 24713005.

14. Remis RS. Modelling the Incidence and Prevalence of Hepatitis C Infection and its Sequelae in Canada, 2007 Public Health Agency of Canada, 2010.

15. Thein HH, Yi Q, Dore GJ, Krahn MD. Estimation of stage-specific fibrosis progression rates in chronic hepatitis C virus infection: a meta-analysis and meta-regression. Hepatology. 2008;48(2):418-31. Epub 2008/06/20. doi: 10.1002/hep.22375. PubMed PMID: 18563841.

16. Alavi M, Raffa JD, Deans GD, Lai C, Krajden M, Dore GJ, et al. Continued low uptake of treatment for hepatitis C virus infection in a large community-based cohort of inner city residents. Liver Int. 2013. Epub 2013/10/30. doi: 10.1111/liv.12370. PubMed PMID: 24164865.

17. Grebely J, Raffa JD, Lai C, Krajden M, Kerr T, Fischer B, et al. Low uptake of treatment for hepatitis C virus infection in a large community-based study of inner city residents. J Viral

Hepat. 2009;16(5):352-8. Epub 2009/02/20. doi: 10.1111/j.1365-2893.2009.01080.x. PubMed PMID: 19226330.

18. Excellence NIfHaC. Peginterferon alfa and Ribavirin for the Treatment of Mild Chronic Hepatitis C. 2006.

19. Soriano V, Peters MG, Zeuzem S. New therapies for hepatitis C virus infection. Clinical infectious diseases : an official publication of the Infectious Diseases Society of America. 2009;48(3):313-20. Epub 2009/01/07. doi: 10.1086/595848. PubMed PMID: 19123867.

20. Wendt A, Adhoute X, Castellani P, Oules V, Ansaldi C, Benali S, et al. Chronic hepatitis C: future treatment. Clin Pharmacol. 2014;6:1-17. Epub 2014/01/29. doi: 10.2147/CPAA.S30338. PubMed PMID: 24470777; PubMed Central PMCID: PMC3891518.

21. Feeney ER, Chung RT. Antiviral treatment of hepatitis C. Bmj. 2014;348:g3308. doi: 10.1136/bmj.g3308. PubMed PMID: 25002352.

22. Urban Health Research Initiative of the British Columbia Centre for Excellence in HIV/AIDS. Drug Situation in Vancouver 2013. 2nd:[Available from: http://www.cfenet.ubc.ca/sites/default/files/uploads/news/releases/war_on_drugs_failing_to_limit_drug_use.pdf.

23. Hezode C, Fontaine H, Dorival C, Larrey D, Zoulim F, Canva V, et al. Triple therapy in treatment-experienced patients with HCV-cirrhosis in a multicentre cohort of the French Early Access Programme (ANRS CO20-CUPIC) - NCT01514890. Journal of hepatology. 2013;59(3):434-41. Epub 2013/05/15. doi: 10.1016/j.jhep.2013.04.035. PubMed PMID: 23669289.

24. Martin NK, Vickerman P, Grebely J, Hellard M, Hutchinson SJ, Lima VD, et al. Hepatitis C virus treatment for prevention among people who inject drugs: Modeling treatment scale-up in the age of direct-acting antivirals. Hepatology. 2013;58(5):1598-609. Epub 2013/04/05. doi: 10.1002/hep.26431. PubMed PMID: 23553643; PubMed Central PMCID: PMC3933734.

25. Kleinman SH, Lelie N, Busch MP. Infectivity of human immunodeficiency virus-1, hepatitis C virus, and hepatitis B virus and risk of transmission by transfusion. Transfusion. 2009;49(11):2454-89. Epub 2009/08/18. doi: 10.1111/j.1537-2995.2009.02322.x. PubMed PMID: 19682345.

26. Hajarizadeh B, Grebely J, Applegate T, Matthews GV, Amin J, Petoumenos K, et al. Dynamics of HCV RNA levels during acute hepatitis C virus infection. J Med Virol. 2014;86(10):1722-9. Epub 2014/07/22. doi: 10.1002/jmv.24010. PubMed PMID: 25042465.

27. Kim C, Kerr T, Li K, Zhang R, Tyndall MW, Montaner JS, et al. Unstable housing and hepatitis C incidence among injection drug users in a Canadian setting. BMC public health. 2009;9:270. Epub 2009/07/31. doi: 10.1186/1471-2458-9-270. PubMed PMID: 19640297; PubMed Central PMCID: PMC2728719.

28. Turner KM, Hutchinson S, Vickerman P, Hope V, Craine N, Palmateer N, et al. The impact of needle and syringe provision and opiate substitution therapy on the incidence of hepatitis C virus in injecting drug users: pooling of UK evidence. Addiction. 2011;106(11):1978-88. Epub 2011/05/28. doi: 10.1111/j.1360-0443.2011.03515.x. PubMed PMID: 21615585.

29. Canada S. Life Tables, Canada, Provinces and Territories - 84-537-X 2013. Available from: http://www.statcan.gc.ca/pub/84-537-x/2013005/tbl-eng.htm.

30. Yu A, Spinelli JJ, Cook DA, Buxton JA, Krajden M. Mortality among British Columbians testing for hepatitis C antibody. BMC public health. 2013;13:291. Epub 2013/04/04. doi: 10.1186/1471-2458-13-291. PubMed PMID: 23547940; PubMed Central PMCID: PMC3626540.

31. Yu Y-W. Mortality among British Columbians testing for hepatitis C antibody, 1992-2004. Vancouver, British Columbia, Canada: University of British Columbia; 2010.

32. Shah NG, Galai N, Celentano DD, Vlahov D, Strathdee SA. Longitudinal predictors of injection cessation and subsequent relapse among a cohort of injection drug users in Baltimore, MD, 1988-2000. Drug and alcohol dependence. 2006;83(2):147-56. doi: 10.1016/j.drugalcdep.2005.11.007. PubMed PMID: 16364568.
